# A 3D-Printable Robotic Gripper Based on Thick Panel Origami

**DOI:** 10.3389/frobt.2021.730227

**Published:** 2021-09-08

**Authors:** Chenying Liu, Perla Maiolino, Zhong You

**Affiliations:** ^1^ Department of Engineering Science, Oxford Robotics Institute, University of Oxford, Oxford, United Kingdom; ^2^ Department of Engineering Science, University of Oxford, Oxford, United Kingdom

**Keywords:** origami gripper, thick panel origami, kinematic modelling, 3D printing, grasping

## Abstract

Origami has been a source of inspiration for the design of robots because it can be easily produced using 2D materials and its motions can be well quantified. However, most applications to date have utilised origami patterns for thin sheet materials with a negligible thickness. If the thickness of the material cannot be neglected, commonly known as the thick panel origami, the creases need to be redesigned. One approach is to place creases either on top or bottom surfaces of a sheet of finite thickness. As a result, spherical linkages in the zero-thickness origami are replaced by spatial linkages in the thick panel one, leading to a reduction in the overall degrees of freedom (DOFs). For instance, a waterbomb pattern for a zero-thickness sheet shows multiple DOFs while its thick panel counterpart has only one DOF, which significantly reduces the complexity of motion control. In this article, we present a robotic gripper derived from a unit that is based on the thick panel six-crease waterbomb origami. Four such units complete the gripper. Kinematically, each unit is a plane-symmetric Bricard linkage, and the gripper can be modelled as an assembly of Bricard linkages, giving it single mobility. A gripper prototype was made using 3D printing technology, and its motion was controlled by a set of tendons tied to a single motor. Detailed kinematic modelling was done, and experiments were carried out to characterise the gripper’s behaviours. The positions of the tips on the gripper, the actuation force on tendons, and the grasping force generated on objects were analysed and measured. The experimental results matched well with the analytical ones, and the repeated tests demonstrate that the concept is viable. Furthermore, we observed that the gripper was also capable of grasping non-symmetrical objects, and such performance is discussed in detail in the paper.

## Introduction

Originating from the Japanese paper art, origami has been widely used in robotic applications to replace conventional linkages. Through rotations about pre-defined creases, origami is capable of large-scale geometrical transformation from a 2D sheet to a 3D object with predictable motions (Rus and Tolley, 2018). It also enables increased design flexibility and a fast but reliable fabrication process, which are particularly important to developing versatile robots (Lee et al., 2017; Li et al., 2017). Among all the robotic applications, there have been quite a few successful attempts at developing origami-inspired grippers. Some origami grippers, simply made from paper folding and/or cutting, have shown remarkable progress in grasping-related tasks compared to traditional rigid robots. They are able to manipulate fragile and/or irregularly shaped objects (Jeong and Lee, 2018), can easily adapt to various shapes and sizes (Phummapooti et al., 2019), and have the potential to be activated by environmental stimuli for autonomous grasping (Tang et al., 2019). Since paper-based origami grippers require tedious manual work to fold and/or cut paper sheets and are also relatively fragile and prone to fatigue caused by repeated folding and unfolding (Jeong and Lee, 2018), novel manufacturing methods, such as silicone casting (Li et al., 2019), laser cutting (Orlofsky et al., 2020; Yang et al., 2021), and 3D printing (Kan et al., 2019; Lee et al., 2020; Liu et al., 2020), have also been introduced in the design process to replace paper origami, thereby further reducing fabrication complexity and enhancing the performance of grippers.

Despite the great potential demonstrated by existing prototypes, it remains challenging to precisely model and control the motions of origami grippers. To date, most grasping applications inspired by origami have been built based on the kinematics of the zero-thickness sheet. Because the material thickness is always neglected, the motions of normal origami grippers are not very accurate. Specifically, the kinematic modelling becomes less accurate when materials of finite thickness are used instead of sheets of negligible thickness. For instance, a 3D-printed origami gripper proposed by Kan et al. (2019) was designed from an origami pattern for zero-thickness sheets, but the actual material thicknesses were 1.8 and 0.6 mm for the rigid panels and flexible creases, respectively, resulting in a discrepancy between the results of the theoretical model and the experiments. This was noted as one of the limitations of the work. In addition, some origami robots have more DOFs due to multiple folds, which makes it difficult to control and carry out motion planning (Rus and Tolley, 2018). Kinematically, zero-thickness origami is commonly modelled as an assembly of spherical linkages (Demaine and O'rourke, 2007), which tends to have more degrees of freedom (DOFs) than those of their kinematically equivalent thick panel counterparts (Chen et al., 2015). For instance, an origami with a six-crease vertex exhibits 3 DOFs (Tachi, 2009) while the number reduces to 1 when it is turned into thick panels by replacing creases with folding lines either on top or at the bottom of panels of finite thicknesses (Chen et al., 2015). The multiple DOFs of an origami gripper based on zero-thickness sheets may require additional synchronisation or actuators to complete the grasping action. Linkages (Phummapooti et al., 2019), pulleys (Jeong and Lee, 2018; Lee et al., 2020), joints with adjustable stiffness (Firouzeh and Paik, 2017), and hybrid actuation methods (Zhakypov et al., 2018; Chen et al., 2021) have been exploited to coordinate and achieve effective grasping motions. These additional actuation requirements may lead to a more complicated design and fabrication process for the origami grippers. An origami-inspired gripper that can be both simply controlled and easily fabricated at the same time would be desirable.

This research aims to develop an origami gripper based on the kinematics of thick panel origami. The design concept has taken the panel thickness into account, which will allow for a precise kinematic model of the gripper’s grasping motion, less affected by the dimension of the folds. In addition, the use of thick panels will reduce the overall DOFs of the origami gripper, which will reduce the complexity of motion control and simplify the fabrication process.

**FIGURE 1:**
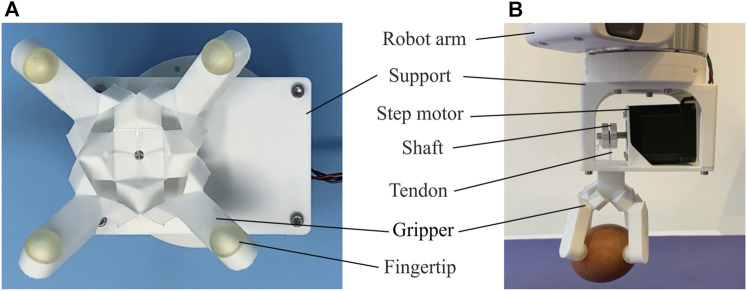
The robotic gripper based on the thick panel waterbomb origami. **(A)** Bottom view of the gripper with four fingers semi-closed. **(B)** Side view of a grasping test on an egg.

In this article, we present a robotic gripper as shown in Figure 1, which is derived from a particular unit that is based on the thick panel waterbomb origami. Four such units are patterned circumferentially to form the backbone of the gripper. Modelled as an assembly of Bricard linkages, the gripper exhibits single mobility. The detailed kinematic modelling was done to predict its grasping trajectories. 3D printing technology was adopted to enable easy and reliable fabrication. The closing and opening motions of the gripper were controlled by a set of tendons that tie to a single motor. Experiments were carried out to characterise the gripper’s behaviours, including its grasping trajectories, the actuation force on tendons, and the contact force generated on objects. With reduced 1-DoF actuation, the gripper was tested on a variety of everyday objects with symmetrical or non-symmetrical shapes. The experimental results match well with the analytical ones, and the repeated tests demonstrate that the concept is viable.

## Materials and Methods

### Waterbomb Unit and its Assembly

**FIGURE 2:**
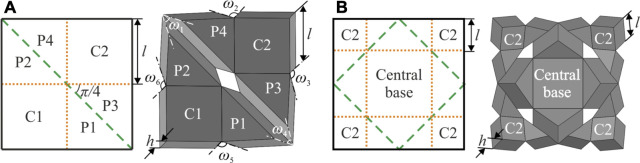
Fold patterns and corresponding 3D kinematic models of **(A)** a single unit and **(B)** its four-unit assembly. Folds on top of blocks are shown by yellow dot lines and at the bottom by green dash lines. Four C1 blocks are patterned circumferentially to form the central base in the assembly and adjacent prisms from different units are merged.

The gripper, displayed in Figure 1, is an assembly of four thick panel origami units. For a better understanding of the mechanism, the fold pattern of a single unit is displayed in Figure 2A, together with its kinematic model. Based on the six-crease waterbomb origami pattern, the unit consists of six blocks: two cuboids, C1 and C2, and four prisms, P1 – P4, which are connected to form a closed kinematic chain by folds either on top or bottom of the blocks. This particular kinematic chain is based on the thick panel origami proposed by Chen et al. (2016).

The waterbomb unit is chosen due to its large deployment ratio and capability to generate circular motions on its corner cuboid (Liu et al., 2020). Both features enable a gripper assembled from such units to mimic the grasping behaviour. Additionally, the unit itself is modelled as a Bricard linkage which has only one DOF. In contrast, its zero-thickness counterpart, the traditional six-crease waterbomb pattern, has six creases meeting at a single vertex, thus leading to 3 DOFs. Therefore, compared to the zero-thickness one, the thick panel unit has the potential to simplify the actuation mechanism and control while achieving desirable motions.

The detailed kinematics of the unit is given below. As shown in Figure 2A, *l* indicates the side length of the cuboid and prisms, and *h* indicates their thickness. Six dihedral angles of the unit, *ω*_1_, *ω*_2_, *ω*_3_, *ω*_4_, *ω*_5_, and *ω*_6_, can be obtained from the following closure loop equations

$$\tan\frac{\omega_{1}}{2}=\frac{\sqrt{2}}{2}\tan\frac{\omega_{2}}{2},\;0<\omega_{1},\;\omega_{2}<\pi$$ (1)

$$\omega_{1}=\omega_{4},\;\omega_{2}=\omega_{3}=\omega_{5}=\omega_{6}$$ (2)

As displayed in Figure 2B, four units are put together to form a gripper by placing four C2 blocks side by side in a circular pattern and merging them to create a central base. The adjacent prisms from different units are combined as well. The C2 blocks retain their ability to complete the circular movement, while the merging steps enable them to achieve mechanical coupling with each other. Therefore, the circular motion preserves its single mobility, and the trajectories of four C2 blocks lead to a wrapping motion when they rotate around the central base. The assembly now forms the backbone of a gripper to wrap around an object for grasping.

### Actuation of the Waterbomb Assembly

**FIGURE 3:**
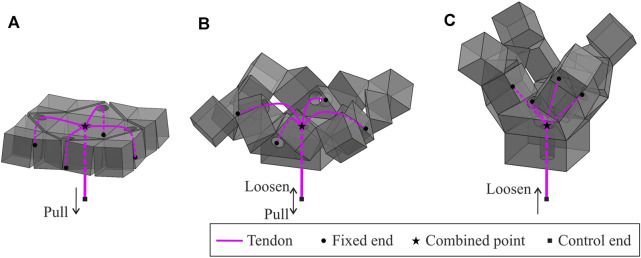
Tendon routings of the assembly and the motions passing through **(A)** the partially folded state, **(B)** the semi-folded state, and **(C)** the near fully folded state. This provides the foundation for grasping behaviour, which is repeatable by pulling and releasing the control end of tendons.

The wrapping behaviour of the assembly is activated by a tendon-based system. As shown in Figure 3, four tendons are fixed on the combined prisms around the central base. Then the tendons pass through the prisms respectively and meet at the centre to be combined for a single control end. The folds connecting the blocks are designed to remain elastic. Consequently, they are only strain-free when the assembly is in the flat state.

Pulling the control end of the tendon in the flat state of the assembly will activate the assembly to perform wrapping. The assembly subsequently passes through the partially folded state, Figure 3A, the semi-folded state, Figure 3B, to the near fully folded state, Figure 3C. This process is defined as the active closing motion of the gripper. When the control end is loosened, the stored strain energy in those elastic folds will be released. As a result, the assembly will gradually return to the strain-free state. This process is the passive opening motion of the gripper. The grasping behaviour is repeatable by pulling and releasing the tendons.

### Gripper Design, Kinematic Modeling, and Grasping Force Analysis

**FIGURE 4:**
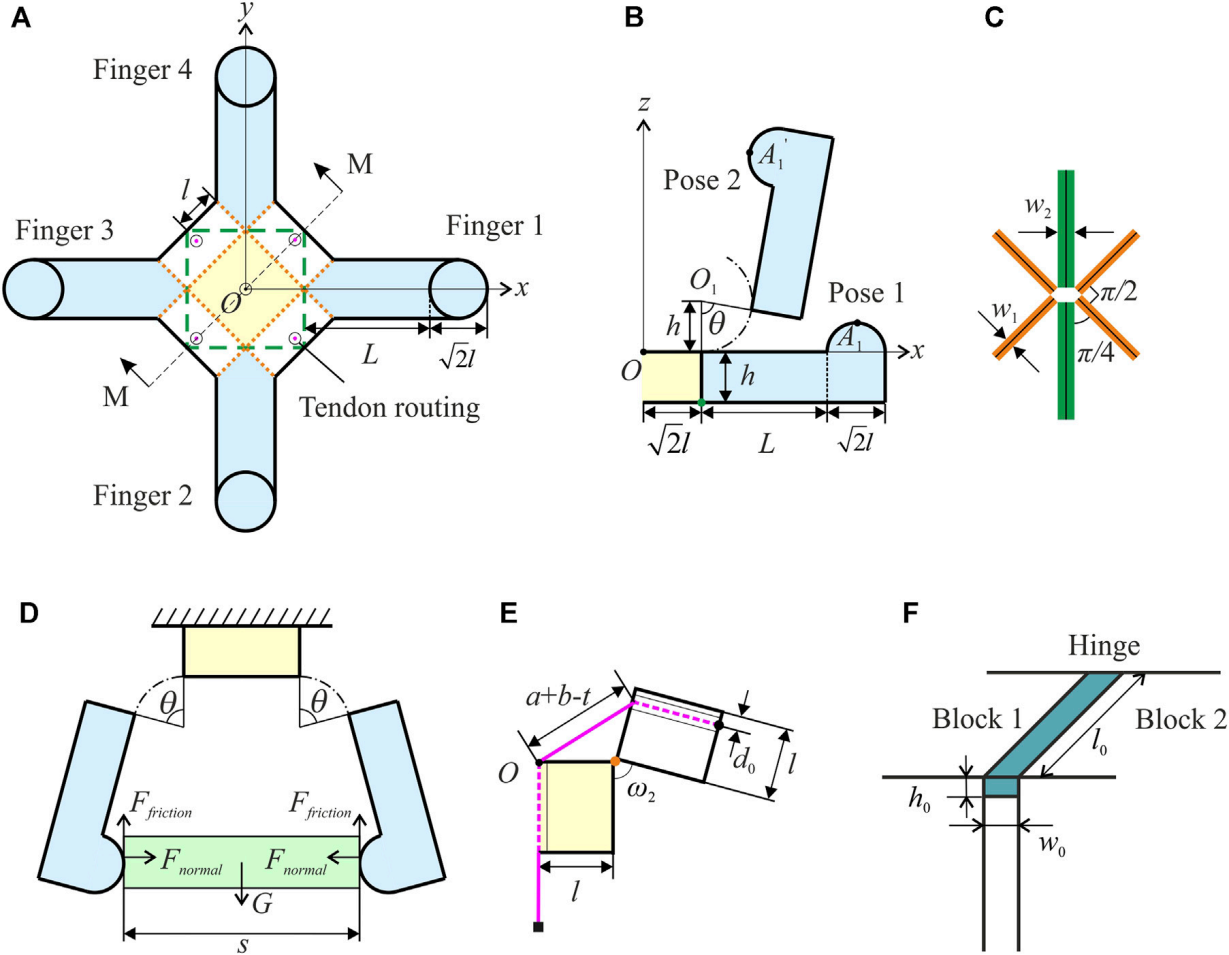
Kinematics of the gripper. **(A)** Proposed design of the origami gripper in the *xOy* plane. Lengthened fingers, fingertips, tendon routings, and folding lines are also denoted. **(B)** Finger 1’s cross-sectional view along the *x* axis at two poses. The closing angle *θ* represents the gripper’s state and the trajectory of a vertex on the finger is depicted using the dot-dash line. **(C)** Details of a vertex where six folding lines meet. The folds’ widths are taken into account and the middle black lines are the new folding creases used to calculate the gripper’s kinematics. **(D)** The gripper’s cross-sectional view along the *x* axis in the grasping mode. Forces applied on the object are indicated. **(E)** Half view of the gripper about section M-M when the gripper is semi-closed. The path of tendons is illustrated. **(F)** Schematic view of a generalised fold in the waterbomb origami together with the parameters to calculate its rotational stiffness.

The four corner cuboids C2 of the assembly serve as the vital component for grasping due to their circular motion trajectories. Therefore, they are regarded as the backbone of four fingers. To enhance grasping capability, these corner cuboids are redesigned to accommodate objects of larger sizes. Figure 4A shows the design of the final gripper at the unfolded state. Corner cuboids are replaced by lengthened fingers whose length is *L*. A hemisphere is added on top of each finger as a fingertip to improve the contact with the objects. Tendon routings and folding lines are marked as well. A cross-sectional view of the gripper at two poses is illustrated in Figure 4B. Due to symmetry, only finger 1 is shown in detail. Pose 1 is the initial flat state of the gripper. The vertex of the finger will rotate about point *O*_1_ with a radius of *h* (Liu et al., 2020). According to the actuation mechanism analysed before, pulling the tendons will activate the four fingers synchronously to pose 2, and even further until the fingertips touch each other. The top centre of each fingertip is used to represent its trajectory. Consider point *A*_1_ of finger 1, whose coordinates are given by

$$x_{A_{1}}=\sqrt{2}l+R\sin\left(\theta +\alpha \right)$$ (3)

$$y_{A_{1}}=0$$ (4)

$$z_{A_{1}}=h-R\cos\left(\theta +\alpha \right)$$ (5)

in the Cartesian coordinate system shown in Figures 4A,B, where $R=\sqrt{{\left(L+\frac{\sqrt{2}}{2}l\right)}^{2}+{\left(h-\frac{\sqrt{2}}{2}l\right)}^{2}}$ and $\alpha =\tan^{-1}\frac{L+\frac{\sqrt{2}}{2}l}{h-\frac{\sqrt{2}}{2}l}$. The closing angle of the gripper, denoted by *θ*, can be obtained from

$$\tan\theta =\frac{2\sqrt{2}\sin\omega_{2}}{1+3\cos\omega_{2}}$$ (6)

Merging Eqs 3, 5 yields

$${\left(x_{A_{1}}-\sqrt{2}l\right)}^{2}+{\left(z_{A_{1}}-h\right)}^{2}=R^{2}$$ (7)

indicating that the fingertip trajectory is also part of a circle with the same centre at point *O*_1_ in the *xOz* plane.

Practically, the widths of the folds may not be negligible due to the fabrication methods. There are two types of folds in the waterbomb unit, one linking two prisms and the other connecting a cuboid and a prism. As illustrated in Figure 4C, *w*_1_ and *w*_2_ indicate the widths of the two types of folds. They will also affect the kinematics of the gripper. The middle black lines marked on those folds are used as the new folding creases. Equations 3, 5, 7 should be rewritten as

$$x_{A_{1}}=\sqrt{2}l+R^{\prime }\sin\left(\theta +\alpha^{\prime }\right)$$ (8)

$$z_{A_{1}}=h-R^{\prime }\cos\left(\theta +\alpha^{\prime }\right)$$ (9)

$${\left(x_{A_{1}}-\sqrt{2}l-\frac{\sqrt{2}}{2}w_{1}-\frac{1}{2}w_{2}\right)}^{2}+{\left(z_{A_{1}}-h\right)}^{2}={R^{\prime }}^{2}$$ (10)

where $R^{\prime }=\sqrt{{\left(L+\frac{\sqrt{2}}{2}l+\frac{\sqrt{2}}{2}w_{1}+\frac{1}{2}w_{2}\right)}^{2}+{\left(h-\frac{\sqrt{2}}{2}l\right)}^{2}}$ and $\alpha^{\prime }=\tan^{-1}\frac{L+\frac{\sqrt{2}}{2}l+\frac{\sqrt{2}}{2}w_{1}+\frac{1}{2}w_{2}}{h-\frac{\sqrt{2}}{2}l}$.

If folds could only have a rotation about the black line at its centre, the gripper would be only suitable to grasp objects with a regular-shaped cross-section such as a square or circle. While grasping such objects, the cross-sectional view of the gripper along the *x* axis is shown in Figure 4D. The object considered here is a cuboid with a square base whose side length is *s*. The relationship between the side length *s* and the closing angle of the gripper *θ* is

$$s=\sqrt{2}l+\sqrt{2}w_{1}+w_{2}+2r\sin\left(\theta +\beta \right)$$ (11)

where $r={\left(L+\frac{\sqrt{2}}{2}l+\frac{\sqrt{2}}{2}w_{1}+\frac{1}{2}w_{2}\right)}^{2}+h^{2}$ and $\beta =\tan^{-1}\frac{L+\frac{\sqrt{2}}{2}l+\frac{\sqrt{2}}{2}w_{1}+\frac{1}{2}w_{2}}{h}.$

According to Eq. 11, the maximum side length of an object that the gripper is able to hold is $\sqrt{2}l+\sqrt{2}w_{1}+w_{2}$ when *θ* is the complementary angle of *β*. Theoretically, the minimum side length is $\left(2-\sqrt{2}\right)l$ when adjacent fingertips touch each other.

While grabbing an object, the contact force between the gripper and the object can be broken down into two components: a normal contact force component *F*_*normal*_ and a frictional force component *F*_*friction*_, which are illustrated in Figure 4D on the object in contact with two fingers. Theoretically, the sum of normal contact forces generated by four fingers is zero while the sum of friction should equal the object’s weight. The coefficient of friction between fingertips and objects plays a crucial role in the grasping process and a larger coefficient leads to a higher maximum grasping weight.

The pulling distance of tendons is the other kinematic aspect to represent the closing state of the gripper. Another cross-sectional view of the gripper is drawn in Figure 4E to show the tendon routing. Assume that tendons are pulled to be straight in the active closing process, and as marked in Figure 4E, the distance between the centre of tendon routing to the edge of the prism is *d*_0_. Take the flat configuration of the gripper as the initial state, and the pulling distance of tendons is given by

$$t=a+b-\sqrt{a^{2}+b^{2}+2ab\cos\omega_{2}}$$ (12)

where $a=l+\frac{1}{2}w_{1}$ and $b=l+\frac{1}{2}w_{1}-d_{0}$. Equations 11, 12 together reveal the relationship between the pulling distance of tendons and the size of the object that the gripper can hold.

The strain energy stored in the gripper’s folds is also analysed to estimate the actuation force required on the tendons. Considering the potential size of the gripper and the weight of 3D printing material, the gravitational force applied to the gripper is regarded insignificant at this stage. Therefore, the potential energy accumulated in the active closing process is the strain energy of the folds. The fold is modelled as a beam, shown in Figure 4F, whose stiffness is given by Hanna et al. (2014)

$$k=\frac{El_{0}h_{0}^{3}}{12w_{0}}$$ (13)

where *E* is Young’s modulus of the fold’s material, and *l*_0_ and *h*_0_ are the length and thickness of the fold, respectively.

A single fold’s strain energy is equal to $0.5k{\left(\omega -\omega_{0}\right)}^{2}$, where $\left(\omega -\omega_{0}\right)$ is the rotational angular displacement of the fold. The stiffnesses of two different folds are defined as *k*_1_ and *k*_2_, respectively. At the strain-free state, all the dihedral angles are zero, that is, the initial values of *ω*_1_ and *ω*_2_ are zero. For a gripper in the active opening process, the strain energy is therefore given by

$$V=4\left(k_{1}\omega_{1}^{2}+2k_{2}\omega_{2}^{2}\right)$$ (14)

The coefficient 4 is due to the fact that the gripper consists of four units and each unit has two *k*_1_ folds and four *k*_2_ folds.

The velocity of the tendons is relatively low during grasping, and the whole system is thus modelled as a quasi-static process. Friction between tendons and the gripper is also considered trivial compared to the strain energy stored in the elastic folds and is thus neglected. Therefore, the work done on the tendons by the pulling force *P* is completely converted into the strain energy, which is given as

$$\int^{\text{​}}P\;dt=V$$ (15)

Therefore, the pulling force *P* applied on the tendons is estimated as

$$P=\frac{dV}{dt}$$ (16)

### Fabrication Process

The gripper design requires: 1) elastic folds to complete repeatable grasping motions; and 2) fingertips with sufficient friction to increase maximum grasping weight. To simplify the fabrication process and make it reliable and replicable, 3D printing was chosen to complete the entire process. The fabrication was carried out in two parts: the first being the backbone structure based on the thick panel origami to perform grasping motions whilst the other being the fingertip coats to increase friction with objects. The detailed process is given below.

**FIGURE 5:**
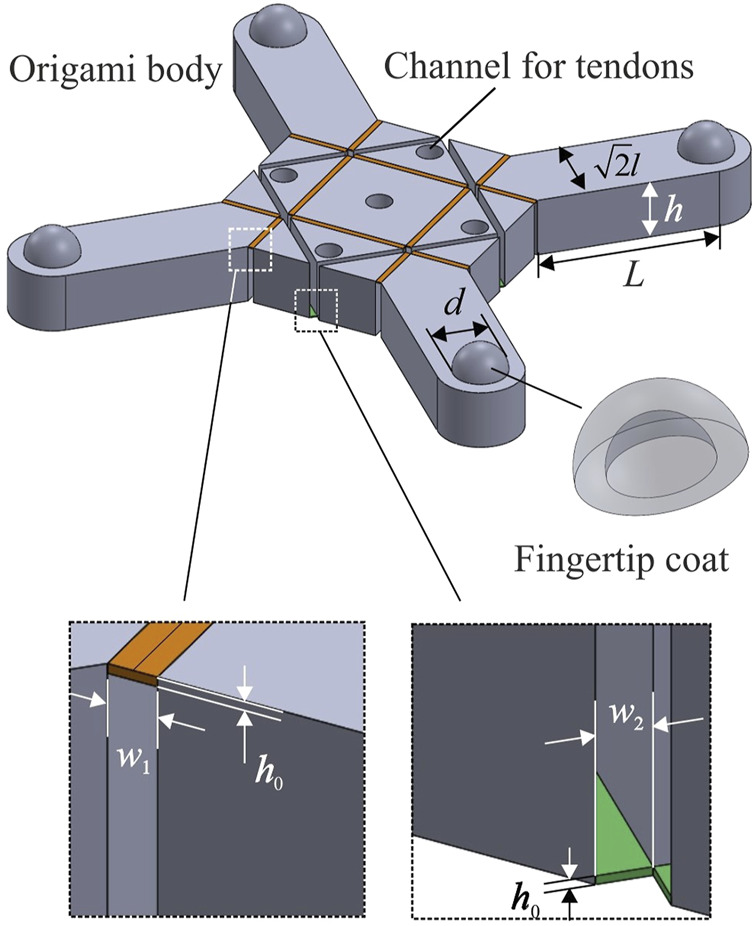
CAD model of the origami body for 3D printing and a schematic view of the fingertip coat. Channels to accommodate tendons and detailed parameters of two folds are also denoted. Note that tiny points with a radius of 0.1 mm were added to the fingertip coat to increase friction before sending it for fabrication.

The computer-aided-design (CAD) model of the origami backbone structure is illustrated in Figure 5. The geometric parameters of blocks were kept the same as the original design while the folds were transformed into thin layers with a width about 0.1 times the side length *l* and a thickness about 0.02 times the thickness *h*. The specific parameters were selected according to the resolution of the used 3D printer. Thermoplastic Polyurethane (TPU) was used as the filament to 3D print rigid blocks and elastic folds in a batch by simply varying their thickness. In particular, the folds’ thickness *h*_0_ was chosen as 0.2 mm while their width *w*_1_ (top folds) and *w*_2_ (bottom folds) were 1 and 1.2 mm, respectively. Both the side length *l* and thickness *h* were set to be 10 mm. The length of a finger *L* was 30 mm and a hemisphere with a diameter *d* of 9 mm was then added on each finger as the fingertip. Five channels were made inside blocks and their diameter was set as 4.3 mm to accommodate tendons and reduce their friction between the blocks. A small cross was placed at the end of each channel excluding the central one to serve as the fixed end of tendons. Empirically, the displacement of the surrounding channel centre to the edge *d*_0_ was set at 4 mm, which showed the most satisfactory performance in actuating the gripper. The fabrication was carried out on a 3D printer (Ultimaker 3 extended) using Shore A hardness 95 TPU filament. The layer resolution was set to be minimum as 0.1 mm.

Figure 5 also shows a schematic view of the fingertip coat whose inner and outer diameters are 9.2 and 10.14 mm, respectively. Tiny points with a radius of 0.1 mm were evenly embossed on the outer surface to increase friction. Four fingertip coats were made using Agilus 30 Clear material on a polyjet multi-material 3D printer (Stratasys J735) with a vertical resolution of 0.14 μm. The fingertip coats were then manually glued onto the fingertips. Clear nylon fishing lines were used as tendons. The gripper weighs 22 g in total.

As shown in Figure 1, the gripper was then mounted on a supporting frame and fixed to a single step motor (NEMA 17). The supporting frame was designed with connection holes so that it is also suitable to be bolted to a robot arm for grasping performance evaluation. The control end of tendons passed through the frame and was tied on the motor’s shaft. An Arduino UNO board was used to control the motor’s movements. Closing and opening motions of the gripper were thus obtained from clockwise and anticlockwise rotations of the motor. The whole fabrication process, including 3D printing and assembling, was completed within 5.5 h without much human intervention.

### Experimental Protocol

Experiments were carried out to characterise the gripper’s behaviours, including its motion trajectories, the actuation force required to close it, the contact force generated on objects, and its performance on grasping everyday objects.

A gripper with fingertip coats that have a 2 mm-high protruding part at the top centre was used for trajectory characterisation. The protruding parts worked as markers whose positions were recorded by a desktop 3D scanner (EinScan Pro+) with a resolution of 0.24 mm. The gripper was gradually opened from the completely closed configuration until it turned into the flat state. Then without much of a pause, the gripper was closed until all the fingertips collided again. This process is considered as a complete cycle of the gripper’s motion. Since the 3D scanner requires the scanned object to be still, a step motor was used to provide a series of still positions. The motor rotated at a 10.8° interval, six times the motor’s minimum step, which provided 43 positions for each fingertip in one cycle. The scanner acquired positions of each fingertip, including 22 in the opening process and 21 in the closing process. The cycle was repeated five times and all the position data were recorded to calculate an average and Standard Error of the Mean (SEM). These data provide a foundation to assess the gripper’s motion trajectory, the difference among four fingers’ performance, and the repeatability of the closing and opening motions.

**FIGURE 6:**
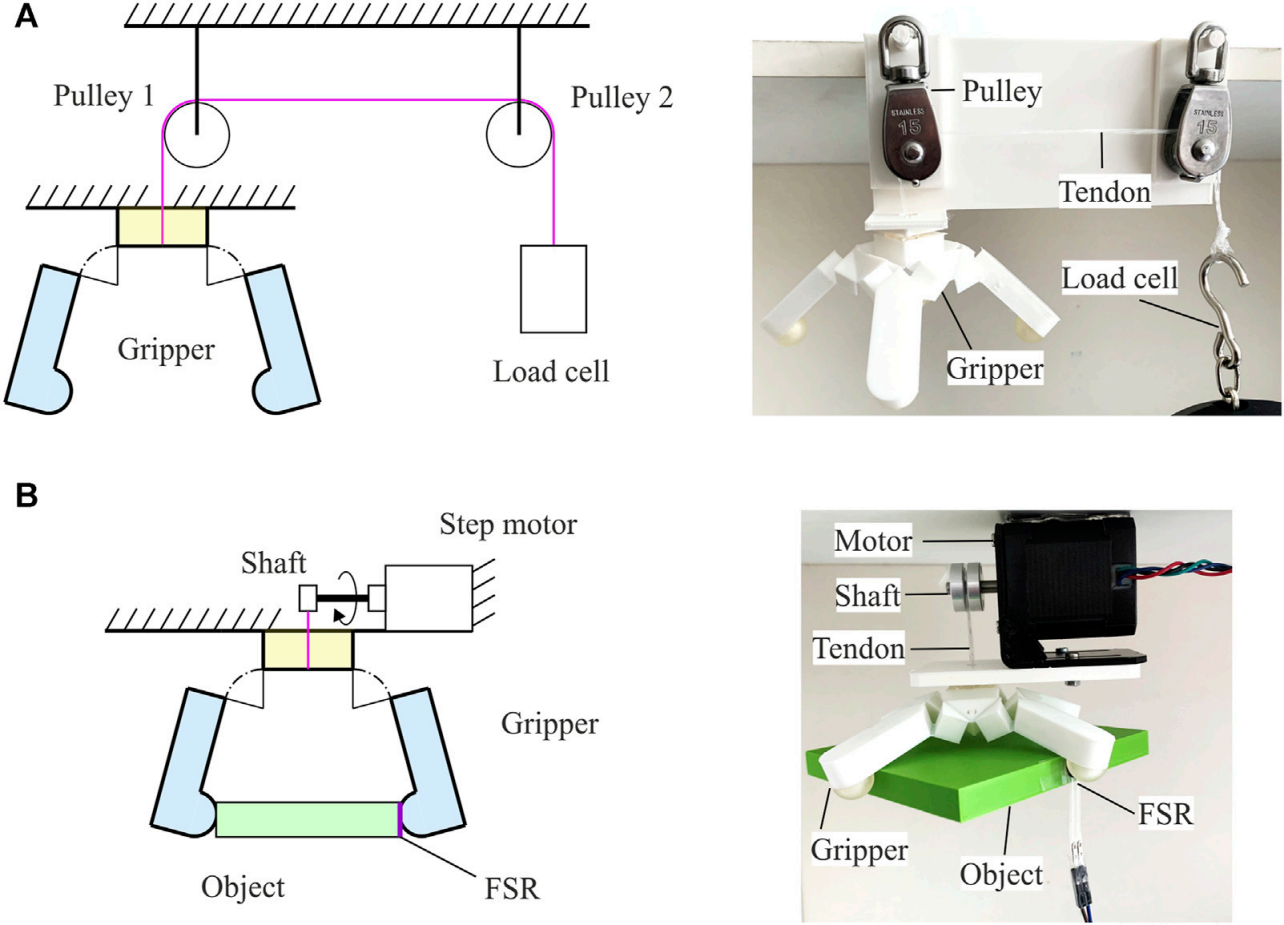
Experimental setup to measure **(A)** the pulling force required to close the gripper to different states and **(B)** the maximum contact force that the gripper is able to generate on objects of different sizes. Both schematic views and practical settings are displayed.

As shown in Figure 6A, to investigate the actuation force required to take the gripper to different states, the gripper was hung on a fixed frame and two pulleys were placed on top of it to guide the tendons, which were further connected to a load cell. The maximum pulling distance of tendons was around the side length of a cuboid component for the gripper, i.e., 10 mm. Based on the kinematics described in Figures 4D,E together, the distance between two opposite fingertips was controlled instead and then converted back to the tendons’ pulling distance by using Eqs 11, 12. The fingertips’ distance was controlled by touching a series of square-based cuboids with side lengths ranging from 10 to 100 mm with a step of 10 mm. The readings of the load cell were recorded six times for each distance and the average was calculated.

The same set of cuboids were used to test the contact force as shown in Figure 6B. The gripper, together with the motor, was fixed on the frame to grasp each cuboid. A force resistive sensor (FSR) with a thin plastic pad to distribute the contact force on the surface was attached to the side of the cuboids. The gripper was closed to its maximum for each cuboid, and 100 continuous readings were taken from the FSR to calculate an average. Calibration of the FSR was performed on a weight scale in advance.

Lastly, the gripper was bolted to a robot arm (PANDA) as shown in Figure 1, and its grasping capability was evaluated on 20 everyday objects of various shapes, sizes, weights, and textures. The test on each object was conducted in the following sequence: 1) lower the robot arm to approach an object placed on a flat surface; 2) close the gripper to grab the object; 3) lift the arm and hold the object for around 10s. Twelve trials were performed on each object, and the number of successful grasping was counted.

## Results and Discussion

### Motion Trajectories of the Gripper

**FIGURE 7:**
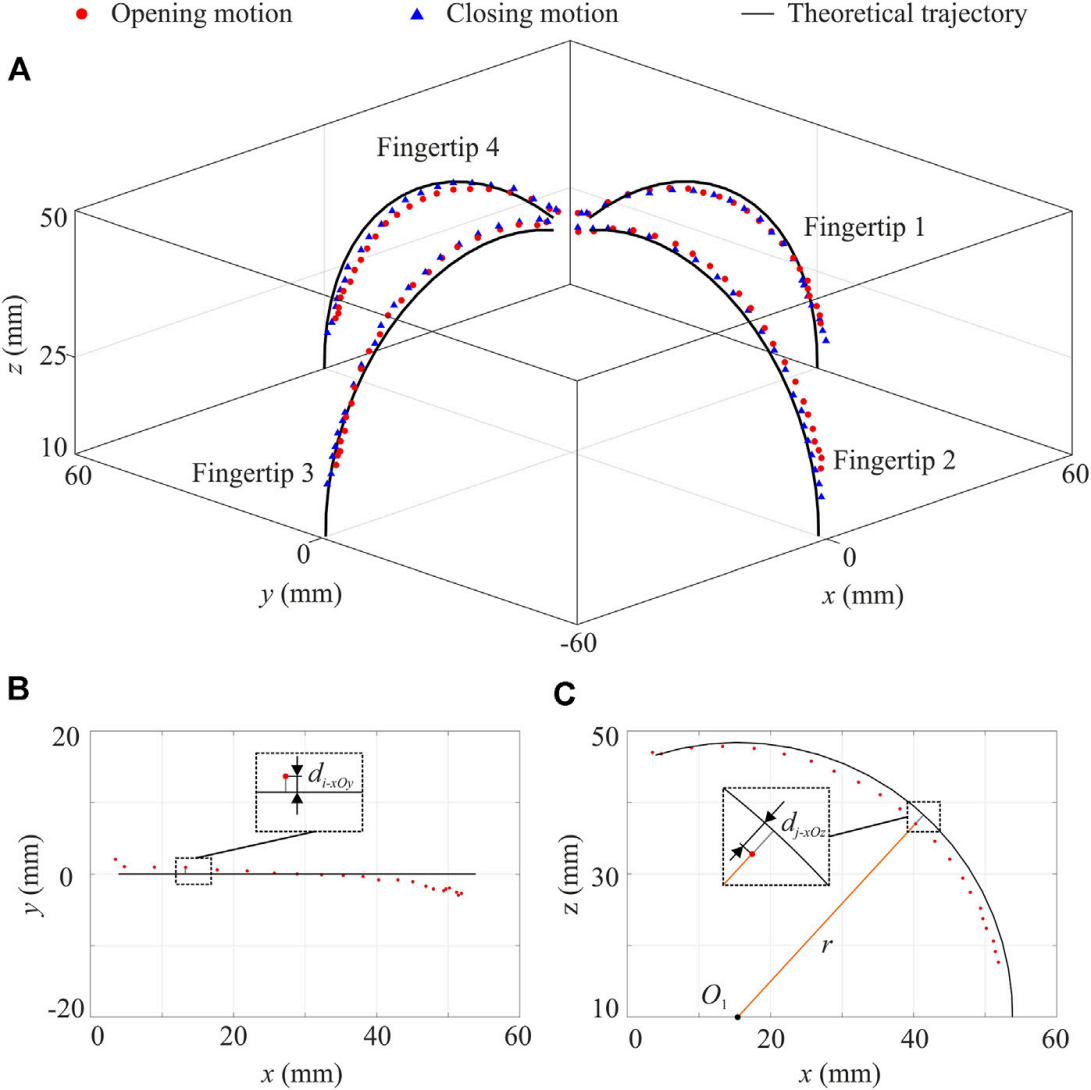
Motion trajectories and position errors of the gripper. **(A)** Experimental results of 3D fingertip positions in the opening and closing motions together with the theoretical trajectories. Calculation of position error in **(B)** the *xOy* plane and **(C)** the *xOz* plane by taking a random position in the opening motion of fingertip 1 as an example. Theoretically, in the *xOy* plane, all positions’ *x*-coordinates (for fingertips 2 and 4) or *y*-coordinates (for fingertips 1 and 3) are zero. Each position error is the absolute value of the position’s *x*-coordinate or *y*-coordinate. The error of fingertip 1’s *i*-th position in the opening motion is plotted as *d*_i_-*xOy*. In the *xOz* plane (for fingertips 1 and 3) or *yOz* plane (for fingertips 2 and 4), the theoretical trajectory of each fingertip is part of a circle. The position error here is thus quantified as the displacement from the circle. The position error of the *j*-th position of fingertip 1 is marked as *d*_j_-*xOz*.

Figure 7A illustrates the positions of each fingertip in the opening and closing motions. The position data used here are the average along three axes from the five repeated experiments. The SEM was calculated at each position, ranging from 0.03 to 1.38 mm, which were considered insignificant compared to the range of these positions. Therefore, the average of each position was then used for the subsequent discussion. As shown in Figure 7A, the theoretical trajectory of each fingertip’s top centre is plotted while the height of the protruding part is also taken into account. To quantify the difference between experimental results and the theoretical predictions, the fingertips’ trajectories are analysed in the 2D planes.

First of all, the experimental opening and closing positions are compared with the theoretical lines, respectively. The opening motion of fingertip 1 is taken as an example. As shown in Figures 7B,C, its 2D trajectories in the *xOy* and *xOz* planes are plotted together with position errors. Detailed 2D trajectories of all fingers in the opening and closing motions are given in the Supplementary material. The maximum position errors of those fingers are summarised in Table 1. As for the position error in the horizontal plane, i.e., the *xOy* plane, fingertip 4 has the best performance in both motions. In the vertical plane, i.e., the *xOz* or *yOz* plane, fingertip 3 has the highest accuracy. The maximum position error within all four fingers is 3 mm. In general, the experimental positions have good conformability with theoretical ones while slight differences are visible when comparing four fingertips. Such behaviours can be attributed to the slightly different material properties in the folding layers caused by the 3D printing fabrication. Such asymmetricity in the gripper has the potential to enable it to grasp objects of irregular-shaped cross-sections in addition to those with a regular shape as discussed before.

**TABLE 1:** Maximum position error (MPE) of fingertips in the 2D plane.

Finger	MPE in the opening motion (mm)		MPE in the closing motion (mm)	
	*xOy* plane	*x(y)Oz* plane	*xOy* plane	*x(y)Oz* plane
1	2.96	1.70	2.60	1.89
2	1.80	3.03	1.89	2.56
3	2.60	1.29	3.00	1.66
4	1.55	3.00	1.03	1.99

The experimental opening and closing trajectories of each fingertip are also compared to understand if there is hysteresis between the two-way motions. This comparison is to quantify the difference between opening and closing trajectories, which theoretically should be zero. In the *xOy* plane, the trajectory displacement is calculated as the difference of their maximum position errors with the theoretical line. As for the trajectory displacement in the *xOz* or *yOz* plane, positions in the experimental opening (or closing) motion for each fingertip are fitted into a circle using the least square method. Then the positions in the experimental closing (or opening) motion are used to calculate their trajectory displacements from the fitted circle, and the maximum value is taken as the trajectory displacement. The data of trajectory displacement are summarised in Table 2. There is a slight difference between the two-way motions, which may be due to the elastic hysteresis behaviour of the TPU-printed folding layers.

**TABLE 2:** Fingertip trajectory displacement between the experimental opening and closing motions in the 2D plane (unit: mm).

Finger	*xOy* plane	*x(y)Oz* plane	
		Fitted closing motion	Fitted opening motion
1	0.36	1.54	0.38
2	0.09	1.37	1.27
3	0.40	0.69	0.78
4	0.52	1.67	1.33

### Pulling Force of Tendons

**FIGURE 8:**
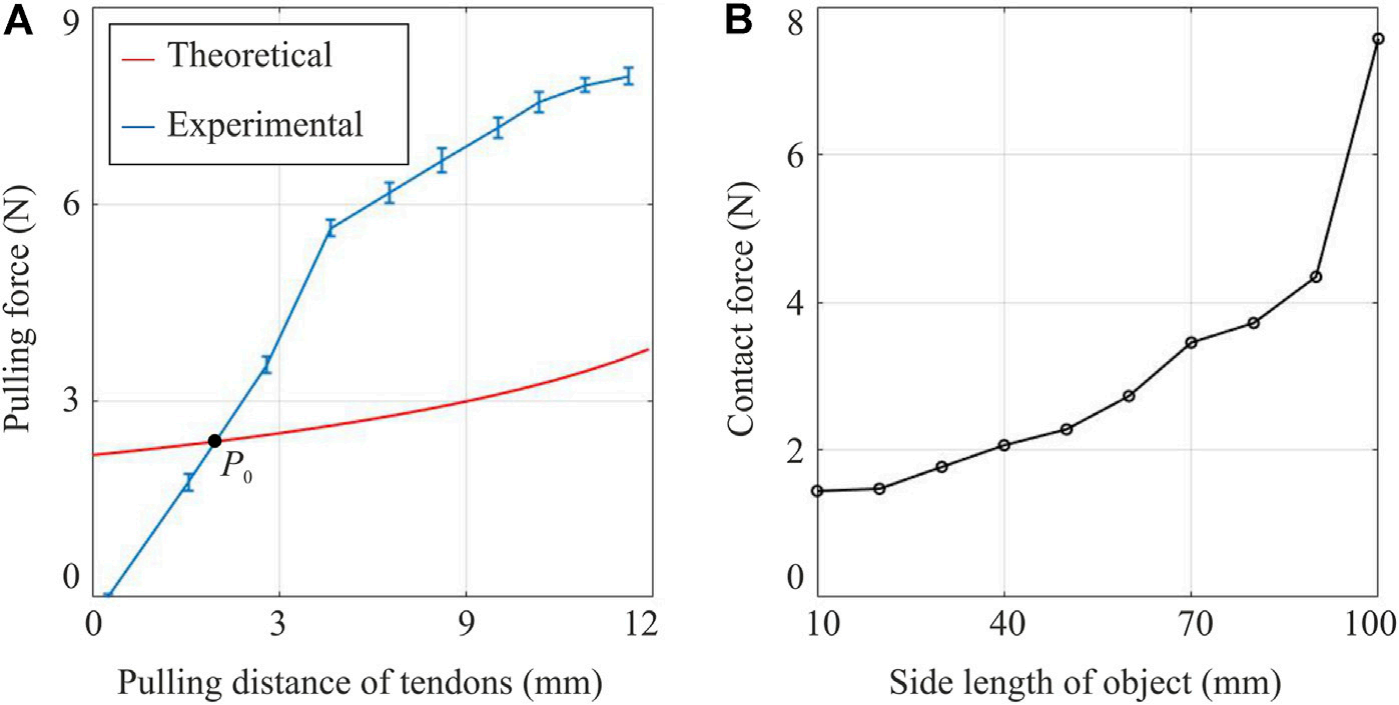
Characterisation results of the gripper’s actuation and contact forces. **(A)** Theoretical and experimental pulling forces on the tendons with error bar *vs*. the pulling distance. The pulling distance of tendons was converted from the side length of the object that the gripper is able to accommodate. **(B)** The contact forces generated on objects of different side lengths, ranging from 10 to 100 mm.

Figure 8A shows the theoretical and experimental pulling forces required to activate the gripper to different states. The pulling distance of tendons was used as the input, and Young’s modulus of TPU, needed to calculate the folds’ stiffness, was measured at room temperature using the dynamic mechanical analysis (DMA) tester. The experimental pulling distance was converted from the size of the object using Eqs 11, 12. The two equations together reveal a negative relationship between the pulling distance and the object’s size, i.e., an object of smaller size requires a longer pulling distance on tendons, and vice versa. As shown in Figure 8A, there is a discrepancy between the experimental pulling force and the theoretical prediction while the two lines intersect at *P*_0_ as marked in the figure. At a small pulling distance on the left side of *P*_0_, i.e., there is a large object to hold, the experimental force is less than the theoretical one. This is because that the gripper’s weight will naturally close itself a bit to hold an object even though there is no pulling force to activate it. However, when holding a smaller object, which means the pulling distance falls on the right side of *P*_0_, the experimental force required surpasses our prediction and the discrepancy between our model and experimental pulling force was as high as 5N. On the one hand, this might be caused by the friction of tendons since they were in contact with the TPU-printed gripper and two pulleys, whose influence was completely neglected in the theoretical analysis. Considering that it is hard to estimate the friction of tendons, one solution is to adopt Polytetrafluoroethylene (PTFE) tubes to guide the tendons, thereby reducing the friction. On the other hand, the elastic folds may also show certain nonlinear behaviour at a relatively large folding angle, which was not represented in the theoretical model. Additionally, it should be noted that when touching objects with various side lengths, the gripper’s maximum grasping size is 100 mm, which is 110.8% of the gripper’s side dimension of 90 mm. This demonstrates that the gripper is even able to accommodate objects larger than its own size. The maximum grasping size is also more significant than the theoretical prediction from Eq. 11, which is 96 mm. The elastic deformation in the folding layers should have played a role in this process.

### Contact Force Generated on Objects

The contact force generated on objects of different sizes is plotted in Figure 8B. The bigger the size of an object, the higher is the contact force generated by the gripper. This can be explained by considering the actuation force on tendons. Since the output torque of the motor remains almost the same, if a lower force is required to activate the gripper, which means a shorter pulling distance on tendons and a bigger size of the object (obtained from Eqs 11, 12), a higher force can also be exerted to close the gripper further to generate contact force. Therefore, the gripper will perform better in grasping a larger object than a smaller one with the same surface texture.

### Evaluation of Grasping Performance

The specifics and the successful grasping rates on the chosen 20 objects are given in Table 3. Selected grasping images of 6 objects are displayed in Figure 9 and a video of the gripper’s performance is available in the Supplementary material. The results indicate that the gripper can grasp objects of regular and irregular shapes, although it was designed for the former. This versatility can be attributed to the grasping trajectories and the elasticity of the folds. Theoretically, the gripper has four circular grasping trajectories evenly distributed on its four fingertips, which enable it to wrap around an object of regular shape. Practically, the four trajectories are not perfectly symmetric with each other, which has been discussed in Figure 7. The elastic deformation inside the folding layers contributes to such asymmetricity, which, in turn, helps with the passive adaptability to irregular-shaped objects.

**TABLE 3:** Everyday objects for grasping performance test and their successful grasping rate.

Object	Weight (g)	Successful rate	Object	Weight (g)	Successful rate
Toilet paper	160	12/12	Data line	27	7/12
Snacks	28	10/12	Toy	87	12/12
Dice	31	12/12	Glue bottle	232	12/12
Hand Sanitizer	60	9/12	Marker pen	14	6/12
Sponge	8	12/12	Force scale	143	11/12
Mini iron	258	8/12	Lucky knot	78	12/12
Mug	524	10/12	Lunchbox bag	171	8/12
Orange	86	11/12	Towel	122	12/12
Egg	60	12/12	Memory stick	20	12/12
Socket	46	8/12	Pulley	41	12/12

**FIGURE 9:**
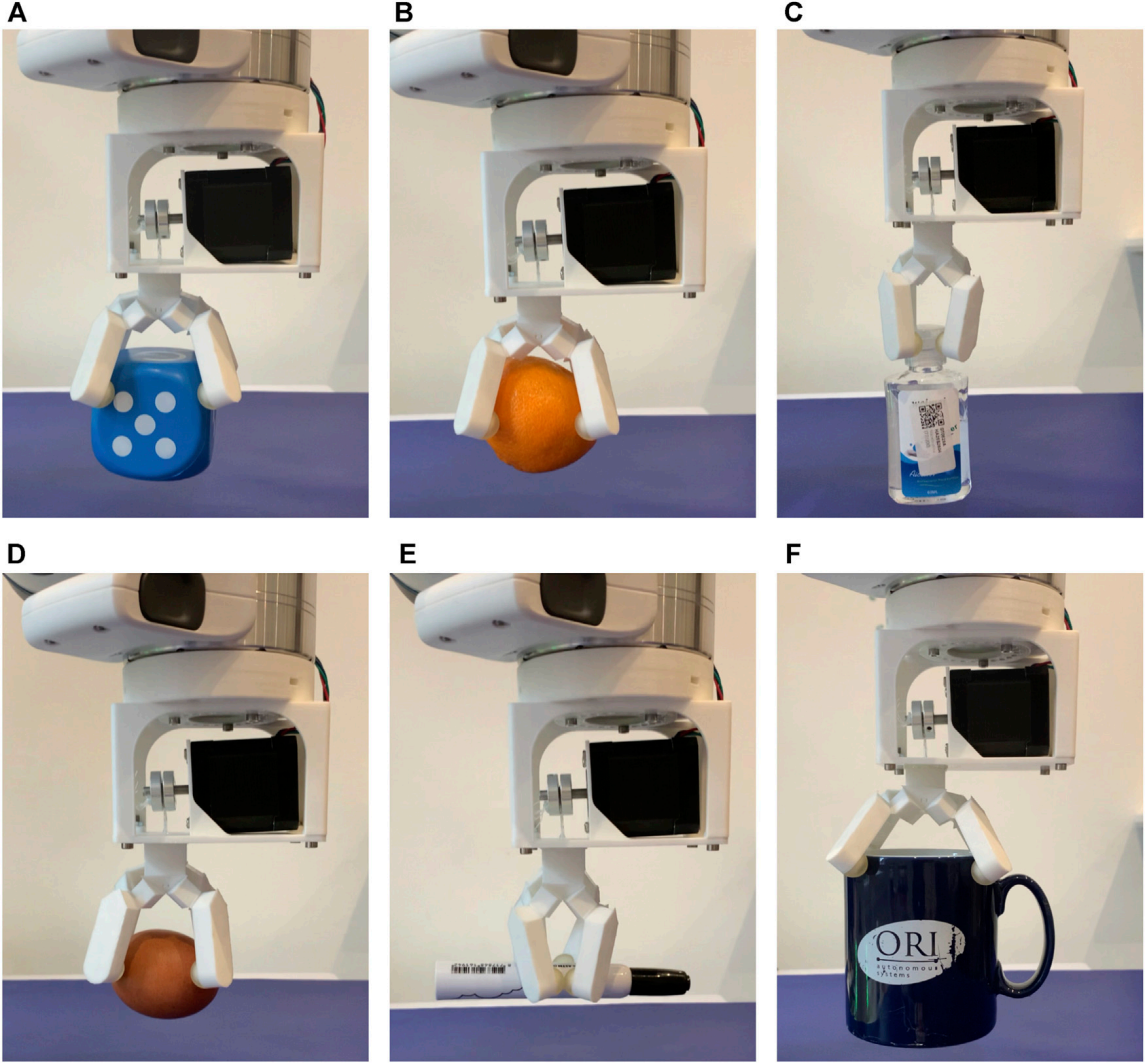
Demonstration of grasping daily objects, which include **(A)** a dice, **(B)** an orange, **(C)** a hand sanitiser, **(D)** an egg, **(E)** a marker pen, and **(F)** a mug with water.

Although the gripper shows relatively stable grasping on selected objects as indicated in Table 3, its performance varies significantly in terms of object size, shape, and texture. For instance, the maximum grasping weight shown in Table 3 is the mug with water inside it, even though it has a relatively low coefficient of friction on the ceramic surface. The mug’s diameter is around 90 mm, close to the maximum grasping size at 100 mm. As discussed in Figure 8, the gripper tends to exert a relatively high normal contact force on an object of such a size, which will increase the maximum stiction with objects and help to improve the grasping performance.

## Conclusions and Future Remarks

In this paper, a single DOF robotic gripper inspired by thick panel origami is created from an assembly of four six-crease waterbomb units. The 3D-printable gripper is able to generate synchronous grasping motions on its four fingers with tendon-based actuation controlled by a single motor. The fingertip trajectories are modelled and also experimentally validated. The gripper can be easily fabricated and it shows good adaptability and relatively stable grasping to objects of different shapes, sizes, weights, and textures. To summarise, 3D printing an origami gripper based on thick panels has largely reduced the fabrication complexity, improved the accuracy with predictable and reliable kinematic behaviour. In addition, the 1-DoF actuation makes motion control significantly simpler.

We also discovered that the discrepancy between predicted and actual tendon forces is partly due to the omission of the frictions between the tendons and their routing channels. One way to overcome this issue is to adopt PTFE tubes to guide the tendons, thus reducing the friction. In the evaluation of grasping performance, it has been observed that the gripper is able to pick up objects of both regular and irregular shapes, which has been largely attributed to its inbuilt compliance within the elastic folds as well as the slight asymmetricity among four fingers to a less extent. This feature could be better utilised through a thorough investigation of the influence of the elasticity of folds on the gripper’s performance, which may well result in the development of a gripper capable of picking up objects of any shape. It is also possible to add vision sensors to the gripper so a feedback control loop can be formed to enable the fingertips to reach a pre-defined position and better adapt to various objects.

## Data Availability

The original contributions presented in the study are included in the article/Supplementary Material, further inquiries can be directed to the corresponding author.
