# Supplementary material for: A 3D-Printable Robotic Gripper Based on Thick Panel Origami
Source: Front Robot AI. 2021 Sep 8;8:730227. doi: 10.3389/frobt.2021.730227 (PMC8455838; doi:10.3389/frobt.2021.730227)
Supplement: Supplementary file 2 [file Image1.pdf]

Supplementary Material

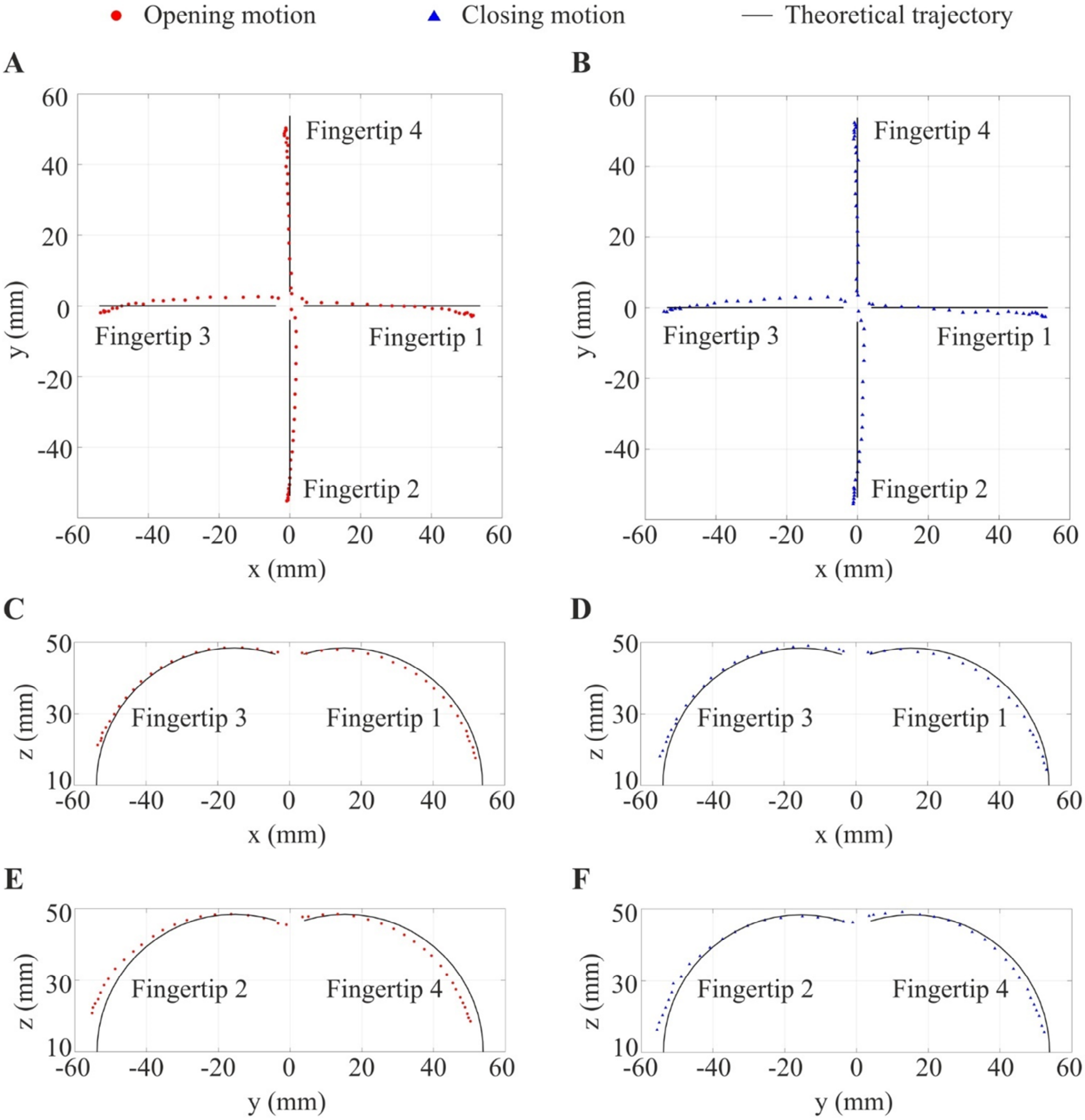

Supplementary Figure 1. Experimental 2D trajectories of all fingertips' **(A)** opening and **(B)** closing motions in the *xOy* plane. Trajectories of fingertips 1 and 3 in the *xOz* plane are given in **(C)** and **(D)**, while the ones for fingertips 2 and 4 in the *yOz* plane are provided in **(E)** and **(F)**. The coordinates are based on the frame displayed in Fig. 4 and the position data correspond to the 3D trajectories in Fig. 7. Theoretical trajectories are put together for a better comparison with the experimental data.
